# Fine-scale foraging movements by fish-eating killer whales (*Orcinus orca*) relate to the vertical distributions and escape responses of salmonid prey (*Oncorhynchus* spp.)

**DOI:** 10.1186/s40462-017-0094-0

**Published:** 2017-02-20

**Authors:** Brianna M. Wright, John K. B. Ford, Graeme M. Ellis, Volker B. Deecke, Ari Daniel Shapiro, Brian C. Battaile, Andrew W. Trites

**Affiliations:** 1 Marine Mammal Research Unit, Institute for the Oceans and Fisheries, University of British Columbia, AERL Building, Room 247 - 2202 Main Mall, Vancouver, BC V6T 1Z4 Canada; 2 Department of Zoology, University of British Columbia, #4200 - 6270 University Blvd., Vancouver, BC V6T 1Z4 Canada; 3 Pacific Biological Station, Fisheries and Oceans Canada, 3190 Hammond Bay Road, Nanaimo, BC V9T 1K6 Canada; 4 Centre for Wildlife Conservation, University of Cumbria, Rydal Road, Ambleside, Cumbria L22 9BB UK; 5 Woods Hole Oceanographic Institution, 266 Woods Hole Road, Woods Hole, MA 02543-1050 USA

**Keywords:** Foraging, Movement, Diving behavior, Biologging, Dtag, Accelerometry, Killer whale, *Orcinus orca*, Pacific salmon

## Abstract

**Background:**

We sought to quantitatively describe the fine-scale foraging behavior of northern resident killer whales (*Orcinus orca*), a population of fish-eating killer whales that feeds almost exclusively on Pacific salmon (*Oncorhynchus* spp.). To reconstruct the underwater movements of these specialist predators, we deployed 34 biologging Dtags on 32 individuals and collected high-resolution, three-dimensional accelerometry and acoustic data. We used the resulting dive paths to compare killer whale foraging behavior to the distributions of different salmonid prey species. Understanding the foraging movements of these threatened predators is important from a conservation standpoint, since prey availability has been identified as a limiting factor in their population dynamics and recovery.

**Results:**

Three-dimensional dive tracks indicated that foraging (*N* = 701) and non-foraging dives (*N* = 10,618) were kinematically distinct (Wilks’ lambda: *λ*_16_ = 0.321, *P* < 0.001). While foraging, killer whales dove deeper, remained submerged longer, swam faster, increased their dive path tortuosity, and rolled their bodies to a greater extent than during other activities. Maximum foraging dive depths reflected the deeper vertical distribution of Chinook (compared to other salmonids) and the tendency of Pacific salmon to evade predators by diving steeply. Kinematic characteristics of prey pursuit by resident killer whales also revealed several other escape strategies employed by salmon attempting to avoid predation, including increased swimming speeds and evasive maneuvering.

**Conclusions:**

High-resolution dive tracks reconstructed using data collected by multi-sensor accelerometer tags found that movements by resident killer whales relate significantly to the vertical distributions and escape responses of their primary prey, Pacific salmon.

**Electronic supplementary material:**

The online version of this article (doi:10.1186/s40462-017-0094-0) contains supplementary material, which is available to authorized users.

## Background

Effective movement patterns during prey searching and capture are critical to the successful acquisition of resources, and are thus a vital component of the foraging behavior of predators. The efficiency of such movements affects an individual’s ability to meet its daily energetic requirements, which in turn has a direct impact on survival and reproduction, ultimately leading to population-level consequences [1, 2]. The ability to accurately describe and quantify the kinematic characteristics of foraging behavior is therefore of great interest to ecologists. Analysis of movement patterns by predators during the pursuit phase of hunting can also shed light on the escape behaviors and predation avoidance strategies employed by prey. However, detailed behavioral studies of movement can be particularly challenging to conduct on large marine predators, such as killer whales and other cetaceans, as these species are typically far-ranging, are only periodically visible at the surface and move within a complex, three-dimensional environment [1, 3, 4].

Most studies of the foraging behavior of fish-eating, or ‘resident’, killer whales in the northeastern Pacific Ocean have been limited to observations of activity visible at the surface [5–7]. Past studies have shown that groups of resident killer whales tend to separate into smaller subgroups that spread out over several square kilometers while hunting, but travel in the same general direction [5]. Dives by individuals in these subgroups are typically asynchronous, and are often characterized by sudden changes of direction, lunges or milling behavior [5]. Surface observations from previous studies noted that foraging whales usually perform sequences of several short dives followed by a longer dive [5]. Capture success during these longer dives can often be determined from the presence of fish scales and flesh in the upper water column after the whale has surfaced [6, 8]. Such physical remains from kills are especially evident when fish are broken up and shared, a behavior that occurs frequently between maternally related individuals [6, 9].

In addition to surface observations, a few foraging studies have deployed time-depth recorders (TDRs) with paddle-wheel swim speed sensors to quantify the diving behavior of resident killer whales [10, 11]. They have shown that dive rate and swim speeds are greater during the day than at night [11]. TDR data have also revealed that resident killer whales spend very little time (2.4%) at depths >30 m, but that these deeper dives are frequently associated with velocity spikes that may indicate fish chases [10]. The utility of TDR tags is limited, however, as they only collect one-dimensional depth profiles and thus cannot address questions of horizontal or three-dimensional movement and space use. TDR data have not been able to adequately describe how and where resident killer whales capture their prey—information that is needed to fully understand their foraging ecology and behavior.

Resident killer whales feed almost exclusively on Pacific salmon (*Oncorhynchus* spp.) for at least half of the year (May to October) and preferentially consume Chinook salmon (*O. tshawytscha*) over other species [6, 8]. Although Chinook is the least abundant salmonid in the whales’ range [12, 13], it accounted for 71.5% of all identified salmon kills (May to December) in a 28-year study of resident killer whale foraging [6]. Resident preference for consuming this prey species does not appear to be influenced by fluctuations in relative Chinook availability [14]. Annual Chinook salmon abundance has been correlated with resident killer whale survival and birth rates [15], and has also been linked to changes in their social connectivity [16, 17]. The ability of resident killer whales to obtain sufficient quantities of Chinook therefore has important consequences for their population growth and social organization. Residents probably target Chinook because their large size and high lipid content make them the most energetically profitable of all Pacific salmon species [18, 19], and because Chinook are available year-round in the coastal waters of North America [6, 12, 20]. Chum salmon (*O. keta*) is the second largest Pacific salmonid and the next most commonly consumed prey species (22.7%) of resident killer whales, and becomes an important food source in September and October [6]. Smaller salmonids, such as coho (*O. kisutch*) and pink (*O. gorbuscha*) salmon, and various groundfish species are occasionally consumed, but do not appear to contribute significantly to the overall diet of these whales [8].

We sought to produce the first quantitative description of fine-scale foraging behavior by fish-eating resident killer whales. We used data from multi-sensor archival tags to reconstruct the three-dimensional movements of individual killer whales during foraging dives and other underwater behaviors that are otherwise impossible to visualize in the wild. We categorized dives based on their kinematic similarities using a multivariate classification technique, with the particular goal of identifying foraging dives. By closely examining the structure of these foraging dives, we could compare killer whale hunting behavior to the vertical distributions of various Pacific salmonids to see if whales targeted the depth ranges typically used by preferred prey. Reconstructing foraging movements also allowed us to identify common escape strategies employed by salmon in response to pursuit by resident killer whales. Our study lays valuable groundwork for future research, as reconstructed dive paths could be used to identify foraging habitat, assess space use, and estimate energy expenditure by individuals from this threatened population [21], the dynamics of which are limited by prey availability [15].

## Methods

### Study area and tagging methodology

**Fig. 1:**
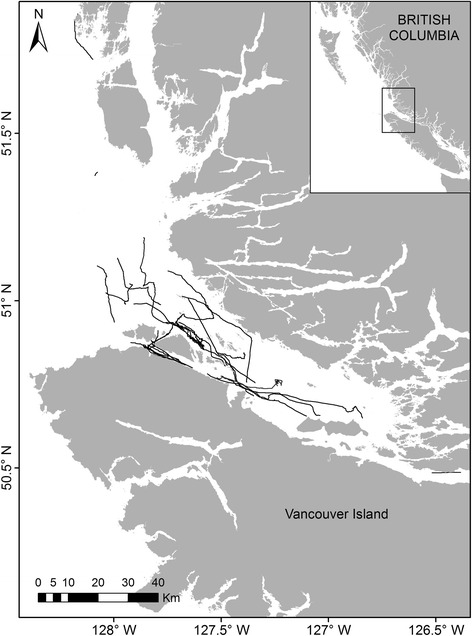
Georeferenced tracks (*black lines*) obtained by dead-reckoning for 31 deployments of archival accelerometry tags (DTags) on northern resident killer whales in British Columbia, Canada during August and September, 2009–2012

We used archival Dtags [22] to record the diving behavior of individuals belonging to the northern resident killer whale community, a population of 290 animals [23] that ranges throughout the coastal waters of the eastern North Pacific, from central Vancouver Island, British Columbia, Canada to southeastern Alaska, USA [24]. Dtags were deployed during August and September (2009–2012) in the coastal waters of northeastern Vancouver Island and the central coast of British Columbia (Fig. 1). The research platform was a 10-m command-bridge vessel powered by a surface-drive propulsion system, which reduced underwater engine noise that could affect the whales’ behavior. When encountered, individual resident killer whales were identified with an existing photo-identification catalogue [23, 25] using a technique developed by Bigg [26]. We then approached an individual by matching its speed and direction of travel and deployed a suction-cup attached Dtag from the bow of the vessel using a 7-m hand-held, carbon-fiber pole. Preferred tag placement was just below the base of the dorsal fin, where the tag’s VHF antenna would clear the water when the whale surfaced, to facilitate tracking of the individual. To minimize potential impacts of tagging, whales were never tagged twice during the same study year (and repeat tagging was avoided whenever possible across study years); we did not deploy tags on juveniles under 3 years of age.

Dtags recorded depth and three-dimensional body orientation (using tri-axial accelerometers and magnetometers) at sampling rates of 50 (2009–2011) or 250 Hz (2012) [22]. They also recorded underwater sound, which helped to identify surfacing events between dives and the timing of prey captures. Surfacing events were characterized by the sound of the tag impacting the air and then the water again as the whale re-submerged, while prey captures coincided with increased flow noise due to body acceleration. Tags detached automatically [22] and were retrieved for downloading of the data. Prior to analysis, sensor data were downsampled to 5 Hz as part of the tag calibration process [22].

### Behavioral observations & prey sampling

We conducted focal follows [27] of tagged individuals and noted surface observations of foraging activity using a digital voice recorder that was time-synchronized with the tag clock. We obtained periodic (mean interval = 21.7 min) GPS surfacing locations throughout each focal follow to apply as positional corrections during tag track reconstruction. GPS fixes were collected with minimal disturbance to the tagged whale by positioning the boat over the ‘flukeprint’ produced after the whale had re-submerged, and matching this location to the associated prior surfacing time (as indicated by a beep from the VHF receiver, recorded on the time-synchronized digital voice-notes). Fluke prints are circular areas of smooth water created from displacement by the whale’s body and turbulence from its tail stroke as it dives, and remain visible on the surface for several minutes after the whale has moved on [28]. The need for concurrent surface observations limited the tag deployments to daylight hours. Following the methodology of Ford and Ellis [6], we collected fish scales and tissue fragments using a fine-meshed dip net when whales surfaced from successful foraging dives. These samples were used to confirm successful predation events and to identify the species and age of the captured fish. Fish species were identified using scale morphology or genetics [29] and schlerochronology was used to establish fish age [30].

### Dtag calibration and identification of dives

Sensor data were calibrated to correct for the orientation of the tag relative to the body axes of each tracked whale, and the raw accelerometer and magnetometer data were converted into pitch, roll, and heading measurements [22]. For some deployments, changes in the position of the Dtag on the animal due to tag slippage required performing new calibrations for every new orientation of the tag. Tag slippage was diagnosed during calibration by looking for abrupt shifts in the central tendencies of the raw accelerometer data, plotted against deployment time. To discount possible reactions to being tagged, we excluded the first 10 min of data for each deployment from further analysis. Most whales displayed mild behavioral responses to tagging (rolling or a slight flinch as the tag was applied) and resumed their pre-tagging swimming patterns within several surfacings (typically <1–2 min).

We identified dives from the calibrated tag data using an automated filter in MATLAB [31] that defined a dive as any event with depth ≥1 m that was bounded by surfacing events of <1 m depth. The shallow depth threshold ensured that all submersions and surfacings were detected. Each surfacing represented a single breath (identified from the acoustic record) and immediate submersion by the tagged animal, although multiple breaths per surfacing (i.e., ‘logging’ behavior, during which the whale remained stationary at the surface) was infrequently noted but discounted from the analysis. We were confident that the MATLAB detection filter estimated the start and end times (relative to time of tag activation) and maximum depth for each dive with high accuracy because we visually compared a random sample of 50 dives against corresponding three-dimensional time-series (or ‘pseudotracks’) of dive behavior that were independently generated using TrackPlot 2.3 software [32]. For 96% of these randomly sampled dives, the times (rounded to the nearest second) and depths (rounded to the nearest 0.1 m) calculated by the MATLAB filter were in agreement with those generated by TrackPlot. Mismatches (>1 s differences) between the MATLAB- and TrackPlot-generated dive times only arose for two dives, which were both very shallow (<2 m) and were bounded by indistinct surfacing events that likely made them difficult for the filter to resolve. We retained these two dives in the analysis because the mismatch in both end times was relatively minor (<3 s).

### GPS-corrected dead-reckoning of tag tracks

We generated a time-series of two-dimensional location data (*x*, *y*) for each whale using dead-reckoning and a MATLAB program (‘*ptrack*’, developed by Woods Hole Oceanographic Institution) that applied a Kalman filter to estimate swim speed from an animal’s pitch and rate of change in depth [22]. These speed estimates were combined with heading measurements to determine the position of each whale relative to its starting location over the length of the deployment. Because dead-reckoning uses estimated prior positions to derive locations farther along the track, absolute position estimates were subject to compounding spatial error over time. To minimize this error, we georeferenced the dead-reckoned tag tracks by constraining them through periodic GPS surfacing (flukeprint) locations that we recorded during the focal follows [33, 34]. GPS ground-truthing of the dead-reckoned tracks reduced the overall error in the time-series of position estimates, although georeferenced tracks with longer time intervals between recorded GPS surfacing locations likely contained greater error than tracks with more frequent fixes [34, 35]. Our GPS-corrected dead-reckoning method also could not entirely account for positional drift of the whale resulting from ocean currents or the influence of forces such as inertia, hydrodynamic lift and buoyancy [36–38]. However, it is important to note that dead-reckoning errors due to either environmental factors or time-dependent cumulative error in estimated speed, pitch or compass heading primarily lead to inaccuracies in the absolute position of tracks [39]. Here, we present comparisons of relative movement over small temporal scales (at the level of the dive) and we employ kinematic variables such as tortuosity that are not impacted by systematic over- or under-estimation of swimming speeds [34]. Dead-reckoning combined with GPS fixes therefore provided a reliable means of producing high-resolution, continuous tracks of underwater movements by tagged whales [33–35, 39]. Georeferenced tag tracks were plotted using ArcGIS software [40] (Fig. 1).

### Calculation of kinematic dive variables

To quantify and compare whale movement patterns, we calculated a set of kinematic variables for each dive using both the raw sensor data and the dead-reckoned whale tracks. These variables included dive duration (s), maximum dive depth (m), two-dimensional dive path tortuosity (i.e., the degree of convolution in the tag track, measured using a straightness index), mean vectorized Dynamic Body Acceleration (VeDBA), maximum absolute roll (degrees), mean absolute roll (degrees), estimated overall dive speed (m s^−1^), and the ratio of descent duration to ascent duration. Additional variables were calculated separately for the descent and ascent phases of each dive: three-dimensional dive path tortuosity, vertical velocity (m s^−1^), mean rate of change in roll (degrees s^−1^), and mean rate of change in pointing angle (degrees s^−1^). We selected the kinematic variables based on their expected ability to distinguish foraging dives from other behaviors. Details concerning the calculation of these kinematic dive variables are presented as Additional file 1 (Appendix A1).

### Multivariate statistical analysis of kinematic dive variables

**Fig. 2:**
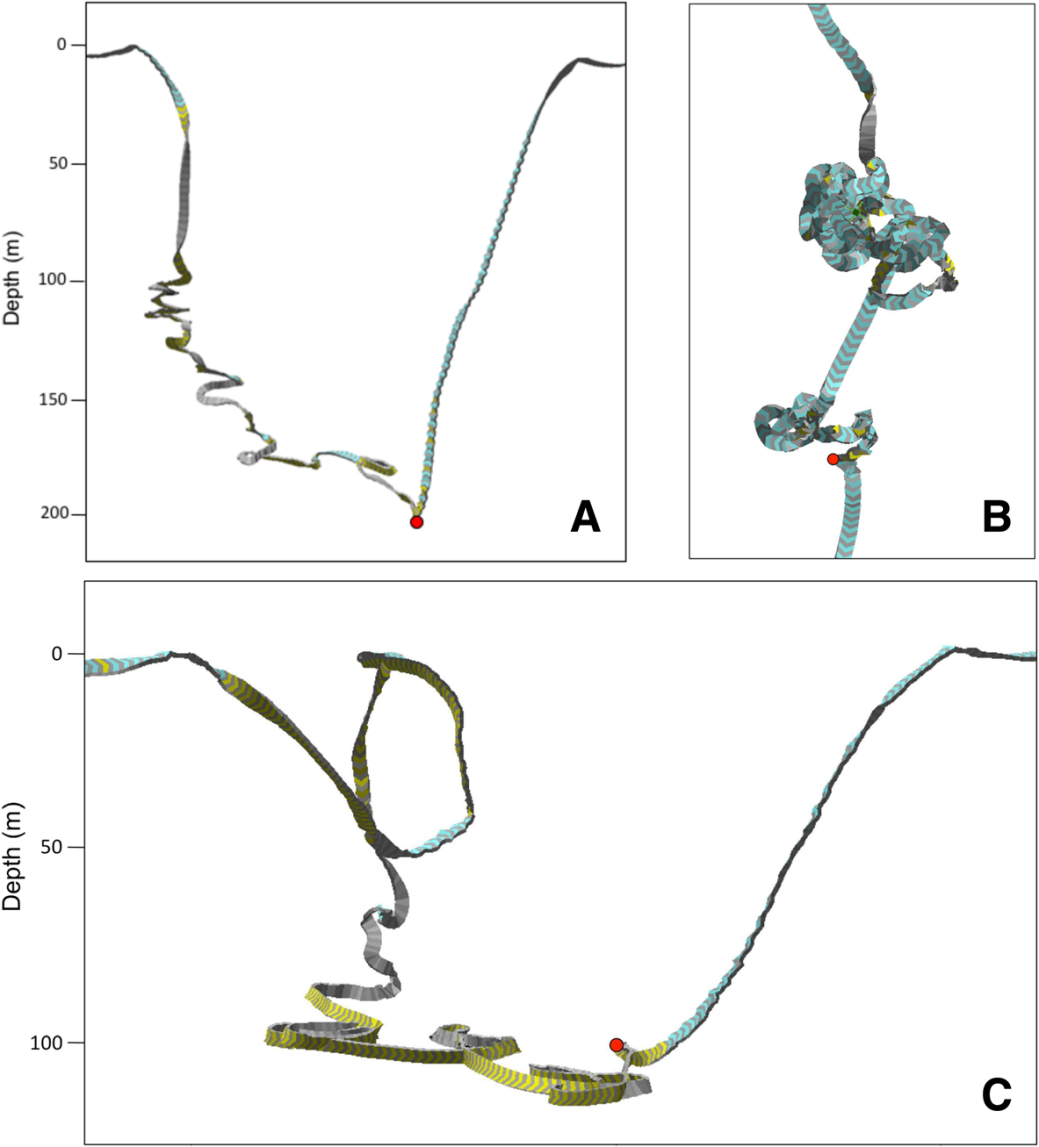
Three-dimensional reconstructions of three foraging dives by northern resident killer whales. Panels (**a**) (V-shaped dive profile) and (**c**) (U-shaped dive profile with maintained ~90° off-axis roll position at the bottom of the dive) are side views of Chinook salmon captures at depth, while (**b**) is an aerial view of a surface chase resulting in a chum salmon capture. *Red dots* represent the probable locations and times of fish captures. *Yellow* portions of the track indicate when the whale rolled sideways >40° in either direction, while blue portions indicate roll <40°

We used the values of the 16 kinematic dive variables measured during successful foraging dives (those from which we obtained fish scale and/or tissue samples, *N* = 17) as the training set in an iterative linear discriminant analysis (LDA) to identify other dives that likely also represented foraging behavior. Two of the confirmed foraging dives were discounted from the LDA training set (leaving *N* = 15 dives), as both of these predation events occurred at the surface, rather than during a dive. Because surface chases were made up of multiple brief, extremely shallow dives (Fig. 2b), the dive-by-dive LDA could only consider very small portions of a surface chase at once, and could not treat all of the dives within the chase as a single capture event.

Prior to performing the LDA, we transformed the kinematic dive variables (except the three measures of tortuosity/straightness) by adding 0.01 to eliminate zeros and then taking the natural logarithm. Since straightness is a proportional measure, the logit transformation was applied to the three tortuosity variables. We added a small value (ε = minimum non-zero value of 1-y; where y represented the range of values of the tortuosity variable being transformed) to both the numerator and denominator of the logit function to prevent proportions equal to 0 or 1 from transforming into undefined values [41]. We assessed whether the data transformations had achieved multivariate normality (an assumption of LDA) by comparing Q-Q plots and histograms of the untransformed versus transformed kinematic variables. We standardized the transformed dive variables by group membership (i.e., the foraging dive training set versus all other unclassified dives) prior to running each iteration of the LDA. Multiple iterations were run in succession, with reassignment of misclassified dives prior to each iteration, until no more dives were detected as misclassified in either category (‘foraging’ or ‘non-foraging’). In every iteration, the 15 confirmed foraging dives with prey samples were always allocated to the ‘foraging’ training set.

Due to the small size of the first training set (*N* = 15) and the small number of whales represented by these dives (*N* = 7), it was possible that idiosyncratic behavior might influence how the LDA identified foraging dives. To determine the relative influence of repeated measures (i.e., the factor of ‘individual’) on the LDA results, we cross-validated the algorithm’s ability to correctly identify foraging dives regardless of within-individual behavior patterns by re-running the analysis with the removal of each whale’s dives in turn from the first training set (‘leave-one-out’ method [42]). This provided a direct test of the LDA’s capacity to correctly classify dives that were not used to calculate the original discriminant function.

Following the iterative LDA, we analyzed the ‘non-foraging’ dives using *X*-means clustering [43, 44] to identify further dive types unrelated to feeding behavior. *X*-means clustering does not rely on *a priori* knowledge of group membership [43], which made it suitable for identifying dive types that lacked ‘true positive’ examples for constructing a training set. Wilks’ lambda tests were performed to determine if the two pairs of dive type groupings, as determined by the LDA (foraging versus non-foraging dives) and *X*-means clustering (various non-foraging dive behaviors), were statistically different from one another. We summarized the untransformed kinematic dive variables by dive type using medians (*M*) and interquartile ranges (IQR), due to the highly skewed distributions of many of these variables.

### Meta-analysis of Pacific salmon vertical distribution

To compare whale diving behavior with that of their prey, we conducted a meta-analysis of the summer and fall vertical distributions of Pacific salmon species. Using reported mean swimming depths from salmon ultrasonic telemetry and tagging studies (*N* = 12), we calculated an overall average swimming depth for each salmon species, which was compared to killer whale foraging dive depths. Where possible, we included mean nocturnal and diurnal swimming depths of tagged salmon as separate values, which allowed the meta-analysis to account for diel variation in depth distribution. If separate day and night values were not available, we used the mean swimming depth for all times of day combined.

We also summarized scientific test fishery studies (*N* = 8) that measured or reported information about the vertical distribution of salmon. We only included studies that reported catch depth for at least 10 individual fish per species. Data from all seasons and times of day were included to ensure that seasonal and diel variations were captured in the analysis. For each salmon species, we determined the depth ranges over which the majority of fish were caught during each study. These species-specific depth ranges were compared to the maximum foraging dive depths of tagged resident killer whales to determine if foraging dives corresponded to the depth range of preferred prey (Chinook salmon).

All studies included in the meta-analysis (both tagging and test fishery) were generally conducted on maturing or adult fish (i.e., those ≥ 2 years old). However, in some cases, fish ages were not specified or studies combined data from juvenile and adult individuals. We did not include studies involving only juvenile salmon (first year at sea) because this age group is not consumed by resident killer whales [6]. To obtain a sufficiently large data set, studies in both coastal and high seas habitats were considered.

## Results

### Tag deployments and dive identification

Dtags were deployed on 34 occasions on 32 different northern resident killer whales (Table 1, Fig. 1). The tagged whales included 8 adult females (≥12 y), 14 adult males (≥12 y), and 10 juveniles (3–11 y; 5 females, 2 males and 3 of unknown sex). Two individuals, A66 and A83, were tagged twice, although the second deployment on A83 was too brief to permit analysis (Table 1). In total, data from three deployments were not analyzed because they had short durations and lacked dives deeper than the 10 m required for calibration. The 31 calibrated tag deployments ranged from 0.3 to 11.8 h in duration, yielding a total of 126.1 h of sensor data (Table 1). The MATLAB dive detection filter identified a total of 11,319 dives (≥1 m).

**Table 1:** Deployments (*N* = 34) of digital archival tags (Dtags) on 32 northern resident killer whales in British Columbia (2009–2012)

Tag ID	Deployment date(dd/mm/yyyy)	Deployment location	Whale ID	Sex	Age (y)	Deployment duration (h)	# dives analyzed
oo09_231a	19/08/2009	50° 46.500 N 127° 24.066 W	G52	F	16	7.41	542
oo09_234a	22/08/2009	50° 56.870 N 127° 47.920 W	A46	M	27	3.92	342
oo09_235a	23/08/2009	50° 49.758 N 127° 43.463 W	A72	F	10	5.22	486
oo09_236a	24/08/2009	50° 51.032 N 127° 31.560 W	I45	M	24	2.37	151
oo09_237a	25/08/2009	50° 47.670 N 127° 31.891 W	I57?	F	20	0.07	0
oo09_237b	25/08/2009	50° 48.336 N 127° 36.855 W	I71	F	16	0.28	0
oo09_237c	25/08/2009	50° 49.336 N 127° 41.669 W	I83	F	12	1.15	93
oo09_237d	25/08/2009	50° 56.672 N 128° 02.190 W	I53	M	23	3.28	314
oo09_238a	26/08/2009	50° 51.117 N 127° 49.327 W	I111	?	3	11.64	1123
oo09_239a	27/08/2009	50° 49.516 N 127° 42.441 W	A66	M	13	2.15	145
oo09_240a	28/08/2009	50° 56.073 N 127° 41.825 W	A37	M	32	3.63	353
oo09_243a	31/08/2009	50° 53.767 N 127° 39.881 W	I39	M	29	3.11	233
oo09_244a	01/09/2009	51° 00.065 N 127° 49.085 W	R25	M	22	4.24	299
oo09_245a	02/09/2009	50° 47.268 N 127° 32.671 W	I46	M	24	5.89	483
oo09_245b	02/09/2009	50° 46.975 N 127° 15.357 W	I62	M	21	1.52	109
oo09_247a	04/09/2009	50° 30.813 N 126° 23.110 W	A62	F	15	1.27	157
oo10_256a	13/09/2010	50° 57.047 N 127° 44.552 W	G64	F	10	7.59	828
oo10_260a	17/09/2010	50° 53.982 N 127° 38.038 W	A75	F	8	6.97	604
oo10_261a	18/09/2010	50° 54.141 N 127° 38.604 W	A38	M	39	3.22	291
oo10_264a	21/09/2010	51° 03.696 N 127° 58.168 W	G39	M	24	1.60	116
oo10_265a	22/09/2010	50° 51.936 N 127° 33.151 W	G49	F	20	2.92	299
oo11_224a	12/08/2011	51° 51.844 N 128° 15.430 W	R40	F	10	2.12	215
oo11_224b	12/08/2011	51° 23.548 N 128° 08.301 W	G32	M	29	0.34	12
oo11_240a	28/08/2011	50° 57.018 N 127° 43.853 W	I104	F	9	3.95	361
oo11_244a	01/09/2011	50° 55.329 N 127° 42.107 W	C14	M	26	2.84	175
oo11_244b	01/09/2011	51° 00.448 N 127° 58.949 W	C24	M	11	1.15	82
oo11_245a	02/09/2011	50° 47.917 N 127° 35.362 W	I43	M	28	11.80	856
oo11_246a	03/09/2011	50° 48.852 N 127° 39.618 W	G31	F	30	3.81	466
oo11_248a	05/09/2011	50° 49.609 N 127° 42.700 W	A83	?	6	0.48	21
oo11_248b	05/09/2011	50° 50.738 N 127° 46.718 W	A80	M	7	2.97	298
oo11_267a	24/09/2011	50° 40.754 N 127° 03.117 W	A34	F	36	7.19	620
oo12_232a	19/08/2012	51° 01.358 N 127° 41.391 W	I106	?	8	5.78	751
oo12_235a	22/08/2012	50° 55.672 N 127° 42.149 W	A83	?	7	0.07	0
oo12_235b	22/08/2012	50° 49.325 N 127° 28.907 W	A66	M	16	4.51	494

Tag IDs reflect the year (e.g., 09) and Julian day (e.g., 231) of tag deployment. Whale IDs, ages and sexes are from published photographic identification catalogues of northern resident killer whales [35, 37]

### Structure of confirmed foraging dives

Prey fragments (fish scales and/or flesh) were collected for 17 confirmed kills that were made by seven of the tagged individuals (Table 2). Scale analysis revealed that nine of these kills were Chinook salmon, six were chum, and two were coho. Salmon caught by the tagged whales ranged in age from 2 to 5 y, with the majority (*N* = 11, 65%) being 4–5 y (Table 2). The pseudotracks for the confirmed foraging dives (with prey samples) revealed a general pattern of convoluted, spiraling and kinematically complex paths during descents, with relatively abrupt transitions (usually at the point of maximum depth) to directional, linear ascents (Fig. 2). Analysis of tag acoustic records suggested that these sudden behavioral transitions likely occurred immediately following prey captures, which allowed us to estimate capture times and depths for successful kills (Table 2). Often, the estimated capture time corresponded with a marked increase in flow noise on the Dtag acoustic record (due to body acceleration) that was followed by crunching sounds (likely indicative of prey processing). A few surface chases were also observed; one chum salmon capture involved only a surface chase (Fig. 2b), whereas four other captures (2 chum, 2 coho) involved surface pursuits followed by a deeper dive that resulted in prey capture. One surface-caught Chinook was taken by a tagged whale (oo12_235b, Table 2) that made a sudden leap at the surface, without any evidence of a pursuit prior to the capture event.

**Table 2:** Summary of confirmed foraging dives (*N* = 17) resulting in fish kills by 7 tagged northern resident killer whales over 4 years (2009–2012) of Dtag deployments

Tag ID	Whale ID	Sex	Age (y)	Date of kill (dd/mm/yyyy)	Capture time (hh:mm:ss)	Capture depth^a^ (m)	Fish species	Fish age^b^ (European)	Fish age (y)
oo09_234a	A46	M	27	22/08/2009	18:46:35	101.6	Chinook	1.1	3
oo09_240a	A37	M	32	28/08/2009	13:02:28	165.7	coho	x.1	≥2
oo09_240a	A37	M	32	28/08/2009	13:29:29	119.4	coho	1.1	3
oo10_256a	G64	F	10	13/09/2010	16:26:52	134.5	chum	0.4	5
oo10_256a	G64	F	10	13/09/2010	16:44:18	123.7 *	chum	0.4	5
oo10_265a	G49	F	20	22/09/2010	17:46:02	130.5	chum	0.4	5
oo10_265a	G49	F	20	22/09/2010	17:53:26	133.7	chum	0.3	4
oo11_246a	G31	F	30	03/09/2011	13:24:46	201.9	Chinook	0.3	4
oo11_246a	G31	F	30	03/09/2011	13:39:04	264.8	Chinook	0.3	4
oo11_246a	G31	F	30	03/09/2011	14:43:15	131.1	Chinook	0.3	4
oo11_246a	G31	F	30	03/09/2011	14:50:32	204.5	Chinook	0.3	4
oo11_246a	G31	F	30	03/09/2011	15:05:47	180.7	Chinook	0.3	4
oo12_232a	I106	?	8	19/08/2012	15:43:54	0.7 †	chum	0.3	4
oo12_232a	I106	?	8	19/08/2012	16:51:35	87.6	chum	0.2	3
oo12_235b	A66	M	16	22/08/2012	14:36:49	102.7 *	Chinook	0.1	2
oo12_235b	A66	M	16	22/08/2012	15:43:56	6.6 *	Chinook	0.2	3
oo12_235b	A66	M	16	22/08/2012	15:57:38	0 †	Chinook	0.3	4

Capture times were determined using a combination of visual (sudden behavioral transitions in the 3-dimensional TrackPlot reconstructions of foraging dives) and acoustic (marked increases in tag hydrophone flow noise due to body acceleration) evidence

^a^Excluding the two surface captures (†), all but three foraging dives (*, maximum depths = 141.4, 103.9 and 32.0 m, respectively) had estimated capture depths that corresponded to the maximum dive depth, as measured by the Dtag pressure sensor

^b^Fish ages are displayed according to the European system, which indicates the number of freshwater and marine annuli (rings) found in the fish scales, separated by a decimal point. Scales for which the number of annuli could not be determined are denoted by an “x” in place of a number

In all but three of the captures at depth (*N* = 15), the probable capture depth corresponded to the maximum depth attained by the whale during the dive (Table 2). Regardless of the salmon species caught, the majority of capture depths (82%) were deeper than 100 m (Table 2). Most of the deeper confirmed foraging dives had V-shaped time-depth profiles (*N* = 11, Fig. 2a). However, a few were U-shaped (*N* = 4) with relatively flat bottom phases accompanied by a sustained body roll of approximately 90° (i.e., individuals swimming on their sides; Fig. 2c). The bottom phases of U-shaped dives also typically contained many tight loops and the whales’ swim paths were more convoluted on average (mean 2D whole dive straightness index = 0.83 ± 0.13 SD, *N* = 4).

### Multivariate statistical analysis of kinematic dive variables

Linear discriminant analysis (LDA) of the 11,319 identified dives detected 701 putative foraging dives over 25 iterations, including the confirmed foraging dives with prey samples used as the initial training set (*N* = 15; two surface captures discounted). The coefficients of the linear discriminant function indicate the weights applied to each kinematic dive variable (Table 3), and variables with larger discriminant coefficients (absolute values) therefore provided the most separation between foraging and non-foraging dive types [45]. In the final iteration (25th) of the discriminant function, the variables that best distinguished foraging from non-foraging dives were dive duration (min), vertical descent velocity (m s^−1^), vertical ascent velocity (m s^−1^), and the ratio of descent to ascent duration (Table 3). Following the LDA, *X*-means clustering split the remaining 10,618 non-foraging dives into two additional types, which we designated as ‘respiration’ (*N* = 7,050) and ‘other’ (*N* = 3,568).

**Table 3:** Median values (*M*) of untransformed kinematic dive variables (interquartile ranges, IQR, shown in parentheses) by dive type, recorded for 30 northern resident killer whales carrying Dtags (31 deployments)

Dive variable	Foraging training set	Foraging dives	Respiration dives	Other dives	Coefficients of linear discriminant
	*N* = 15	*N* = 701	*N* = 7050	*N* = 3568	
Dive duration (min)	3.68 (2.35)	2.94 (2.36)	0.33 (0.27)	0.34 (0.29)	−1.7075
Maximum dive depth (m)	133.67 (61.57)	34.00 (71.02)	2.75 (1.34)	3.15 (1.92)	0.2537
2D dive straightness index	0.86 (0.26)	0.95 (0.12)	1.00 (0.004)	0.97 (0.05)	−0.1045
3D descent straightness index	0.81 (0.16)	0.94 (0.12)	0.99 (0.02)	0.93 (0.10)	−0.1066
3D ascent straightness index	0.90 (0.09)	0.93 (0.11)	0.99 (0.02)	0.93 (0.07)	0.0362
Mean VeDBA	0.17 (0.06)	0.10 (0.07)	0.09 (0.05)	0.11 (0.08)	0.1421
Maximum absolute roll (deg)	179.84 (0.15)	132.32 (128.43)	14.12 (20.72)	28.75 (32.24)	−0.1204
Mean absolute roll (deg)	65.92 (29.17)	21.58 (41.32)	5.50 (7.12)	9.71 (10.55)	−0.0770
Overall swim speed (m s^−1^)	2.72 (0.45)	2.08 (1.12)	1.91 (0.90)	1.17 (0.75)	0.0801
Descent : ascent duration	1.41 (1.31)	0.85 (0.95)	1.05 (0.53)	1.03 (0.61)	−5.1212
Vertical descent velocity (m s^−1^)	0.98 (0.72)	0.66 (0.66)	0.29 (0.15)	0.32 (0.17)	−5.9038
Vertical ascent velocity (m s^−1^)	1.93 (1.02)	0.57 (0.76)	0.30 (0.16)	0.33 (0.20)	4.9175
Descent Δroll/time (deg s−1)	27.08 (13.47)	8.56 (13.57)	4.93 (5.40)	7.34 (6.85)	0.1164
Ascent Δroll/time (deg s−1)	43.52 (26.17)	9.70 (12.55)	4.11 (4.18)	6.45 (5.69)	0.1094
Descent Δpointing angle/time (deg s−1)	57.36 (22.44)	22.62 (29.98)	21.50 (20.53)	26.35 (34.65)	0.0352
Ascent Δpointing angle/time (deg s−1)	51.64 (23.02)	22.05 (21.65)	11.24 (8.52)	15.37 (12.48)	−0.2077

Coefficients of the linear discriminant indicate weights applied to each dive variable, with larger absolute values indicating variables that provided greater separation between the foraging and non-foraging dive types

**Fig. 3:**
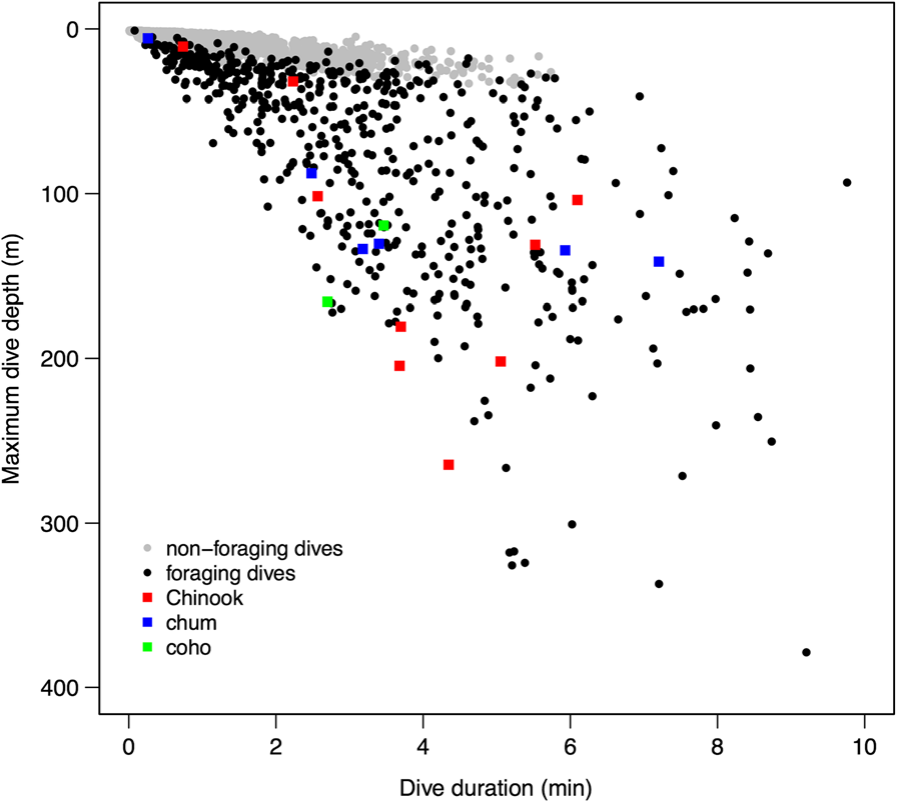
Maximum dive depths (m) and dive durations (min) of foraging (*N* = 701) and non-foraging (*N* = 10,618) dives by 30 tagged northern resident killer whales (number of deployments = 31). Confirmed foraging dives (*N* = 17) are marked by coloured data points indicating the species of salmon killed (Chinook, coho or chum). Non-foraging dives (gray data points) did not exceed 21 m in depth

**Fig. 4:**
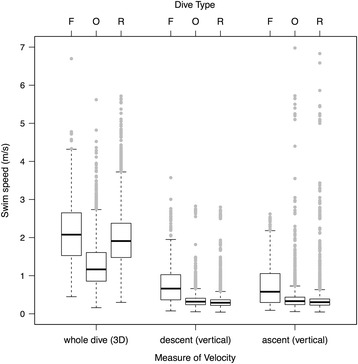
Comparative swim speeds between the three identified dive types made by 30 tagged northern resident killer whales (F = foraging, R = respiration, O = other behaviors; *N* = 11,319 total dives from 31 tag deployments). Whole dive velocity was calculated by dividing the 3-dimensional dive path length (determined using dead-reckoning) by the total dive time, and included both descent and ascent phases. Vertical velocities for descent and ascent phases were based solely on depth sensor data

**Fig. 5:**
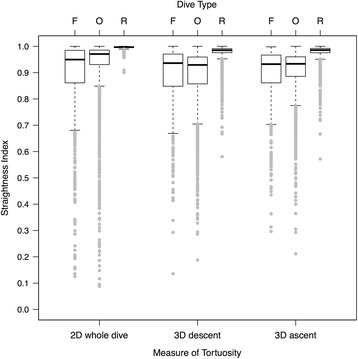
Comparative kinematic tortuosity variables between the three identified dive types made by 30 tagged northern resident killer whales (F = foraging, R = respiration, O = other behaviors; *N* = 11,319 total dives from 31 tag deployments). The straightness index, indicating relative tortuosity, was calculated in two dimensions (x-y plane only) over entire dives and in three dimensions for the descent and ascent phases. Lower values of the straightness index indicate more convoluted paths of whale movement, while values approaching 1 indicate directional, straight-line paths

**Fig. 6:**
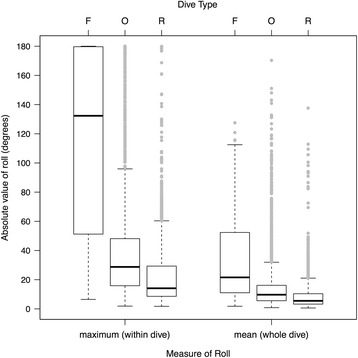
Comparative maximum and mean body roll (absolute values, in degrees) by 30 tagged northern resident killer whales engaged in three identified dive types (F = foraging, R = respiration, O = other behaviors; *N* = 11,319 total dives from 31 tag deployments)

Compared to other dive types, foraging dives identified by the LDA (*N* = 701) were typically deeper (*M* = 34.0 m, IQR = 71.0 m; Fig. 3) and lasted longer (*M* = 2.9 min, IQR = 2.4 min; Fig. 3). Foraging whales also swam at greater estimated speeds (*M* = 2.1 m s^−1^, IQR = 1.1 m s^−1^) than they did during ‘other’ dives, but displayed no difference in speed compared to respiration dives (Fig. 4, Table 3). Foraging dive rates of descent (*M* = 0.7 m s^−1^, IQR = 0.7 m s^−1^) and ascent (*M* = 0.6 m s^−1^, IQR = 0.8 m s^−1^), measured as vertical velocities, were considerably faster than they were for non-foraging dives (Fig. 4, Table 3). Straightness indices in both two (whole dive) and three dimensions (descent and ascent phases) for putative foraging dives (*M* = 0.93–0.95) were marginally lower than those of respiration dives (*M* = 0.99–1.00), indicating that whale movement paths were more convoluted and less directional (i.e., had higher tortuosity) during foraging (Fig. 5, Table 3). Confirmed foraging dives had even lower straightness indices, particularly during the descent phase (*M* = 0.81). However, median straightness values (*M* = 0.93–0.97) for other dive behaviors were similar to those displayed during putative foraging dives (Table 3). Whales engaged in foraging dives also rolled to a greater extent than during non-foraging dives (Fig. 6). Medians of both the mean body roll (*M* = 21.6°, IQR = 41.3°) and maximum body roll (*M* = 132.3°, IQR = 128.4°) values recorded within each dive were considerably higher during foraging dives (Table 3). The summary statistics for the LDA foraging training set (*N* = 15) indicated an even stronger kinematic differentiation from the non-foraging dive categories (Table 3). The confirmed foraging dives in the training set had much greater durations (*M* = 3.7 min, IQR = 2.4 min), depths (*M* = 133.7 m, IQR = 61.6 m), mean (*M* = 65.9°, IQR = 29.2°) and maximum (*M* = 179.8°, IQR = 0.2°) body roll values, overall swim speeds (*M* = 2.7 m s^−1^, IQR = 0.5 m s^−1^), and vertical velocities (descent: *M* = 1.0 m s^−1^, IQR = 0.7 m s^−1^, ascent: *M* = 1.9 m s^−1^, IQR = 1.0 m s^−1^), as well as lower straightness indices (*M* = 0.81–0.90; Table 3).

Non-foraging ‘respiration’ dives identified by *X*-means clustering were extremely shallow (*M* = 2.8 m, IQR = 1.3 m), comparatively brief in duration (*M* = 0.3 min, IQR = 0.3 min), and while only slightly slower than foraging dives in terms of overall speed (*M* = 1.9 m s^−1^, IQR = 0.9 m s^−1^), they had considerably slower median vertical descent and ascent velocities (*M* = 0.3 m s^−1^, IQR = 0.2 m s^−1^; Fig. 4, Table 3). Movement within these dives was highly directional, with almost no tortuosity (*M* ≥ 0.99 for all 3 straightness index measures, Fig. 5) and limited mean (*M* = 5.5°, IQR = 7.2°) and maximum (*M* = 14.1°, IQR = 20.7°) body roll (Fig. 6, Table 3). The kinematics of this dive type corresponded well with surface observations of whales submersing themselves for extremely brief periods between single breaths, a movement that occurs repeatedly between deeper dives and is present during all activity states (e.g., resting, foraging, travelling and socializing). While not really a true ‘dive’, these surface breathing bouts are conducted for the sole purpose of gas exchange during forward propulsion [11], and so we refer to them throughout as ‘respiration dives’, primarily for convenience.

The second type of non-foraging dive was designated as ‘other’ because the overall kinematic structure was intermediate between foraging and respiration dives. Like respiration dives, these dives were comparatively shallow (*M* = 3.2 m, IQR = 1.9 m) and short in duration (*M* = 0.3 min, IQR = 0.3 min). Overall dive speed (*M* = 1.2 m s^−1^, IQR = 0.8 m s^−1^), as well as descent and ascent vertical velocities (*M* = 0.3 m s^−1^, IQR = 0.2 m s^−1^), were almost identical to those of respiration dives and were slower than during foraging dives (Fig. 4, Table 3). However, ‘other’ dive behaviors had straightness indices that were more similar to those of foraging dives and indicated slightly higher path tortuosity (*M* = 0.93–0.97; Fig. 5, Table 3). During ‘other’ dive behaviors, killer whales also exhibited a higher level of mean (*M* = 9.7°, IQR = 10.6°) and maximum (*M* = 28.8°, IQR = 32.2°) body roll than for respiration dives, although not to the same extent as during foraging dives (Fig. 6, Table 3). Given the large number of dives in this category (*N* = 3,568) and the intermediate values of many of the kinematic dive variables (Table 3), it probably includes a variety of other previously described behaviors by resident killer whales, such as socializing, travelling, resting and beach rubbing [5]. *X*-means clustering was likely unable to further separate the different behaviors within this category because the kinematic predictor variables were chosen specifically for their expected ability to identify foraging dives.

All three dive types (foraging, respiration, and other) were detected in all but one of the 31 tag deployments; one short deployment (0.34 h) on the adult male G32 (Table 1) contained no foraging dives. As a percentage of recorded dives (number of dives, not time-budget) for each individual, foraging dives made up an average of 6.7% (SD = 3.5%), while respiration dives comprised 64.7% (SD = 21.0%) and other dive behaviors 28.6% (SD = 20.0%). The considerably higher occurrence of respiration ‘dives’ was likely because they represent bouts of surface breathing that are present throughout all killer whale activity states. Kinematic characteristics of foraging and non-foraging dives detected by the LDA were significantly different (Wilks’ lambda: *λ*_16_ = 0.321, *p* < 0.001), as were the kinematic characteristics of two non-foraging dive types (respiration, other) detected by *X*-means clustering (Wilks’ lambda: *λ*_16_ = 0.323, *p* < 0.001). The majority of variance in the kinematic dive variables (~68%) can thus be attributed to the grouping factor, meaning that both LDA and *X*-means clustering distinguished dive types that differed significantly in their kinematic structures. The non-independence of samples (dives), due to the temporal autocorrelation inherent in time-series data, means that the level of significance implied by the Wilks’ lambda *P*-values is likely somewhat inflated. However, the LDA separated dive types consistently (even when reduced training sets were used during leave-one-out validations), suggesting that within-group (dive type) variance is much lower than between-group variance, and that the dive types can be consistently differentiated from one another. The leave-one-out validations also confirmed that the LDA was not unduly influenced by idiosyncratic variations in foraging dive structure, since all of the omitted individual’s successful foraging dives (with prey samples) that had been excluded from the training set were reclassified as ‘foraging’ by the final iteration of each validation test.

Some of the kinematic variables used in the LDA did not distinguish foraging from non-foraging dives as well as we had expected. Vectorized Dynamic Body Acceleration (VeDBA) was very similar between foraging dives, respiration dives, and other dive behaviors (Table 3). Rates of change in both body roll and pointing angle (descents and ascents; degree s^−1^) tended to be similar between foraging dives and other behaviors, but were generally lower for respiration dives (Table 3). The ratio of descent to ascent durations was expected to be higher (>1.0) for foraging dives, on the basis that descents involving tortuous chase behavior should take longer than directional ascents covering the same depth range. This variable also had a higher absolute value for its linear discriminant coefficient (Table 3), which implies that it was relatively important in predicting group membership (i.e., dive type). Although descent to ascent duration was greater for the LDA training set of confirmed foraging dives (*M* = 1.41, IQR = 1.31), it was actually lower (<1.0) for the putative foraging dives (*M* = 0.85, IQR = 0.95) than for other behaviors (*M* = 1.03, IQR = 0.61), and respiration dives (*M* = 1.05, IQR = 0.53) (Table 3). Overall, however, the IQRs for this variable across the three dive types indicate that the distribution of values for ascent:descent duration is basically equivalent regardless of dive type.

### Meta-analysis of pacific salmon vertical distribution

**Fig. 7:**
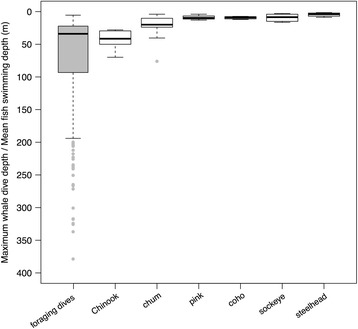
Maximum depths (m) of foraging dives (*N* = 701) by 30 tagged northern resident killer whales (*grey box plot*) and overall mean ocean swimming depths (*white box plots*) of six species of Pacific salmon, as reported in tagging and ultrasonic telemetry studies (*N* = 12) of maturing or adult fish (≥2 years) in summer or autumn

**Fig. 8:**
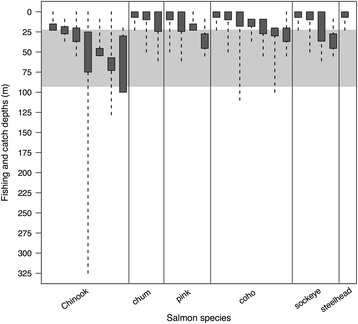
Catch depths (m) of six species of Pacific salmon taken by troll, gillnet or trawl fishing (*dark grey boxes*), and maximum foraging dive depths (1st–3rd quartiles, shaded band) of 30 tagged northern resident killer whales. The range of maximum foraging depths shown here spans the interval between the 25th and 75th percentiles (22.2–93.2 m) of all LDA-detected foraging dives (*N* = 701), and the fishery catch depths are from salmon vertical distribution and bycatch studies. For each species of salmon, individual boxes represent separate studies (some studies appear more than once if conducted on multiple species). Dashed lines indicate the total depth interval (m) fished, and dark grey boxes represent the depth interval (m) in which the largest percentage of fish was caught during each study. Catch data are from all seasons and times of day, taken in both coastal and high seas habitats (*N* = 8 studies, minimum of 10 individual fish/species/study)

The meta-analysis of ultrasonic telemetry and archival tagging studies showed that Chinook salmon swim at an average depth of 43.4 m (SD = 15.4 m, Fig. 7) in coastal and offshore Pacific waters [46–49]. In contrast, chum salmon swim at an average depth of 22.0 m (SD = 19.0 m), while coho ($\overline{x}$ ± SD = 9.4 ± 2.2 m), sockeye (9.4 ± 6.1 m), pink (9.0 ± 3.7 m) and steelhead (4.6 ± 3.2 m) are surface-oriented species found at average depths of less than 10 m (Fig. 7) [48–57]. The meta-analysis of test fishery studies indicated similar patterns of vertical distribution, with most Chinook being caught below 30 m (range = 15–100 m) [49, 58–62], while all other salmon species tended to be caught at depths shallower than 30 m (range = 0–45.5 m) (Fig. 8) [58–64]. The maximum foraging dive depths of tagged northern resident killer whales (*M* = 34.0 m, IQR = 71.0 m, *N* = 701) overlapped considerably (Figs. 7 & 8) with the average vertical distribution of Chinook, but did not correspond well to the swimming depths of other salmon species.

## Discussion

Analysis of dives by northern resident killer whales revealed that dive depth, path tortuosity, body rotation and estimates of velocity are reliable metrics for distinguishing foraging from non-foraging behavior. Most notably, Dtag-recorded kinematics showed that foraging dives by northern residents attained and often exceeded the expected depth distribution of Chinook salmon, their preferred prey. Analyzing whale movement patterns during prey pursuit also revealed several strategies that salmon may use to escape air-breathing predators.

### Kinematic structure of foraging dives

Foraging dives by resident killer whales were characterized by greater maximum depths and dive durations, more convoluted dive paths, higher levels of body rotation, and increased swimming speeds. The median maximum depth (133.7 m, *IQR* = 61.6 m) of confirmed foraging dives (training set, *N* = 15) in our study corresponded with the average maximum depth (calculated per tag) of 140.8 (±61.8 SD) m reported by Baird et al. [11] for southern resident killer whales. The variability in maximum dive depths was also remarkable similar between the two studies. Median durations for both LDA-identified (2.9 min, *IQR* = 2.4) and confirmed (3.7 min, *IQR* = 2.4) foraging dives from the Dtag data were marginally greater than the mean daytime dive durations measured by TDRs deployed on southern residents (2.8 ± 0.5 for adult males, 2.1 ± 0.6 for adult females) [11]. However, the study by Baird et al. [11] pooled all dives ≥1 min together regardless of activity state, which may help to explain this difference.

The increased roll and greater tortuosity displayed during the descent phase of foraging dives by resident killer whales (Fig. 2a) may serve to facilitate acoustic searching. Odontocetes have narrow, conically-shaped sonar beams that allow them to effectively discriminate the size and distance of detected targets [65]. However, to initially locate prey, an area much larger than the whale’s beam width must be scanned. Rolling behavior may increase the area covered by the sonar beam and thus improve the likelihood of detecting prey. Akamatsu et al. [65] found that finless porpoises (*Neophocaena phocaenoides*) rolled extensively during dives with greater acoustic search effort and DeRuiter et al. [66] found that in captive harbor porpoises (*Phocoena phocoena*), both click rate and variance in roll angle increased around the time of fish capture. Similar increases in rolling behavior were prevalent in the foraging dives made by the tagged resident killer whales in our study (Table 3, Fig. 6), and could serve the same purpose. Increased body rotation may therefore be a useful metric for identifying foraging behavior in future studies.

Sustained off-axis body roll positions performed by hunting killer whales during U-shaped dives may also improve maneuverability and swimming performance during fish pursuits along the sea floor. Cetaceans generate hydrodynamic thrust for swimming by moving the posterior third of their bodies and tail flukes dorso-ventrally [67]; the average vertical amplitude of fluke tip movement during swimming by killer whales is greater than 20% of their body length [68, 69]. Tail stroke amplitude would therefore be restricted when moving closely along the sea floor in an upright position. In our study, tagged animals often rotated their bodies approximately 90° to the right or left during the level bottom phases of foraging dives with U-shaped profiles (Fig. 2c). If whales were chasing salmon along the sea floor, as this dive shape implies, then turning sideways would allow unrestricted fluke movement and ensure that high swimming speeds were achieved. Although whales swimming sideways along the bottom may lose the additional thrust and propulsive efficiency generated by ground effect [70], the benefits of unimpeded fluke movement likely outweigh this cost, because the flukes must be very close (within one span length) to the sea floor for ground effect to be of consequence [71].

In addition to depth and tortuosity, estimated swimming velocity was also an effective way to identify foraging dives. Based on dead-reckoned tracks, the median estimated speed of foraging whales in our study was 2.1 m s^−1^ (*N* = 701; Table 3, Fig. 4). However, compared to all foraging dives combined, dives resulting in successful kills (*N* = 15) were slightly faster (*M* = 2.7 m s^−1^) and several foraging dives had estimated speeds exceeding 4.0 m s^−1^ (max = 6.7 m s^−1^; Fig. 4). The somewhat slower median speed of LDA-identified foraging dives, relative to those dives resulting in confirmed kills, is likely due to this dive category also containing unsuccessful, aborted chases (Table 3). Using theodolite techniques, Williams & Noren [72] estimated maximum swimming speeds for adult resident killer whales of 2.7 m s^−1^ (females) and 3.0 m s^−1^ (males), which is similar to the median speed of 2.7 m s^−1^ for our confirmed foraging dives. Estimated speeds for the fastest foraging dives (>4.0 m s^−1^) recorded in our study are likewise comparable to the average maximum velocity of 5.98 m s^−1^ recorded by Fish [68] for captive killer whales performing turning maneuvers. They are also similar to the theoretical sustainable aerobic swimming speed of 4.7-5.6 m s^−1^ estimated for adult killer whales by Guinet et al. [73]. Mean sustained swimming speed for killer whales chasing bluefin tuna (*Thunnus thynnus*) in the Strait of Gibraltar was 3.7 ± 0.2 m s^−1^ [73], which unsurprisingly is slightly faster than the median speed of northern resident killer whales in our study, which were hunting moderately slower-moving Pacific salmon. Roos et al. [74] estimated a mean swimming speed of 1.89 ± 0.61 m s^−1^ (range = 0.69–4.05 m s^−1^) for Norwegian herring-feeding killer whales based on low frequency flow noise from Dtag hydrophone recordings. This mean swimming speed for the Norwegian whales is comparable to our median foraging dive speed for northern residents (2.1 m s^−1^), while the maximum speed is similar to the maximum speeds of the fastest foraging dives in our Dtag dataset.

Estimated swimming speeds were calculated using dive path lengths that relied on dead-reckoning, and therefore contained cumulative error that may have led to the over- or underestimation of distances travelled [34], thus impacting speed calculations. However, we minimized such errors by correcting track placement using periodic GPS surfacing locations, and by constraining our analysis to comparisons of kinematic metrics summarized over very brief time periods (i.e., dive-by-dive). Swim speed estimates from our Dtag tracks concurred with swimming speeds from other studies of fish-eating killer whales that were obtained using different methodologies, and therefore we are reasonably confident of their accuracy. Unlike estimated overall swimming speed, vertical velocities likely contained minimal error because they were calculated directly from depth sensor measurements; however, these values greatly underestimate true swimming speeds because movement in the horizontal plane was not considered. Regardless, both overall swimming speed and median vertical velocity were much higher for foraging dives than for any other dive type (Table 3). Vertical velocities were equally high for both the descent and ascent phases of foraging dives (Table 3)–due to the pursuit of rapidly fleeing prey during descents, and the need to return to the surface quickly during ascents to replenish oxygen stores depleted at depth. Hydrophone records from ascent phases of confirmed foraging dives typically contained pulses of flow noise caused by fluke strokes as the animal ascended, which substantiated depth sensor-detected increases in vertical velocity.

### Foraging dive depth selectivity and the vertical distribution of preferred prey

We determined that the maximum depths of foraging dives by northern resident killer whales overlapped with the average swimming depth of Chinook salmon tracked during tagging studies (Fig. 7), as well as with test fishery catch depths (15–100 m) for Chinook (Fig. 8). Conversely, there was almost no correspondence between maximum dive depths of foraging whales and the average swimming depths of other salmon species (Fig. 7), except for chum, which is the second most commonly consumed prey species by northern residents [6]. This overlap between maximum foraging dive depths (i.e., estimated fish capture depths) and the vertical distributions of Chinook and chum salmon suggests that resident killer whales are intentionally diving to depths where preferred prey is more likely to occur. Although the vertical distribution of salmon changes on seasonal and diel scales, and is affected by many physiological and ecological factors [46, 49, 75], tagging and fisheries studies consistently indicated that Chinook salmon are located deeper in the water column than other salmonids. This means that when Chinook salmon abundance is low, killer whales may continue to dive to the deeper depths used by their preferred prey, but would experience low encounter rates and poor energetic returns.

Although the foraging dive depths of killer whales overlapped with the vertical distribution of Chinook salmon, whales also extended their foraging dives to much greater depths of up to 379 m (Figs. 3 & 7). Chinook have been intercepted as bycatch by trawlers at depths of 325 [49] and 482 m [76]. Ultrasonic tracking has also shown that Chinook salmon swim to depths of 300–400 m, and that fish performing deep dives (>200 m) are significantly larger ($\overline{x}$ = 87.2 cm) than those remaining at shallower depths ($\overline{x}$ = 77.3 cm) [46]. These deep-diving individuals correspond in length to 4–5 y Chinook [77], which are the size classes most frequently consumed by resident killer whales [6]. This suggests that whales may dive beyond the average swimming depth of most Chinook to increase their chance of locating the larger and more energetically profitable 4–5 y old fish.

### Predation avoidance strategies of pacific salmon

Killer whale foraging dives would also be expected to exceed the typical swimming depth of Chinook if salmon swim towards the sea floor as an escape response. The maximum depths of successful foraging dives were greater (Table 3) than the average depth that Chinook are found at (Figs. 7 & 8), implying that whales may chase Chinook to greater depths before catching them. Furthermore, tortuous dive paths that resembled chases occurred primarily during the descent phase for 12 of the 15 successful foraging dives (Table 2), with the capture point corresponding to the maximum depth of the dive (e.g., Fig. 2a). This occurred regardless of the species of salmon caught, suggesting that rapid descents may be a general predator avoidance response of many Pacific salmon. The other three confirmed foraging dives had capture points that did not correspond to maximum dive depths (Table 2). Of these, two were U-shaped dives that had the same high track tortuosity evident in the descent phases of the other 12 dives (Fig. 2c). The level bottom phases of these two dives suggest the whales were chasing fish along the sea floor and the fish could not escape to deeper water, which could explain why the estimated capture points for these dives did not correspond to the maximum dive depths.

Most of the confirmed kills of chum and coho (*N* = 7 out of 8), which are normally shallow-swimming species (Fig. 7), had estimated capture depths exceeding 80 m (Table 2, Fig. 3). While shallow water chases preceded the captures of coho and chum for five of these eight (63%) successful foraging dives, only one chum capture actually occurred near the surface (Table 2). This also indicates that rapid descent is likely a common escape response in all species of Pacific salmon. Foraging whales may have opportunistically encountered chum and coho close to the surface, where they are typically found, and subsequently pursued them to greater depths before making a successful capture. An underwater video (Ellis GM, Towers JR, Ford JKB: 'A' pod juveniles with chum salmon, unpublished media) further supports the hypothesis that surface-oriented salmon species will dive when threatened by a predator. This video shows two young whales echolocating on a chum salmon, which then dives towards the bottom after one of the whales bites its caudal fin. Tagging studies of Pacific salmon provide additional evidence that diving steeply could represent an escape response, as fish often performed very deep dives immediately after post-tagging release [46, 52, 55–57, 78–80]. In addition, tagged chum salmon dove to the sea floor in 12 of 16 encounters with Dall’s porpoises (*Phocoenoides dalli*), a potential predator [81].

Although fleeing is energetically costly [82], swimming downward may be an effective strategy for fish to escape an air-breathing predator, such as a killer whale. The likelihood that a pursuing whale would have to return to the surface to breathe before intercepting its prey would increase with greater dive depths (i.e., longer pursuit times). Salmon may also descend to avoid presenting the visual target of a dark body silhouetted against light coming from the surface [83]. Lastly, swimming to the sea floor could allow fish to use rocky crevices and other bathymetric features as refuges from large predators.

Since Pacific salmon appear to descend rapidly as an escape response, determining depths at which chases are initiated would provide a better estimate than the maximum foraging dive depths we used here (Fig. 7) for identifying the depth ranges targeted by whales searching for prey. This would involve determining the depth of the transition between the search and pursuit phase of each foraging dive, which could be accomplished by combining kinematic and acoustic analyses. The beginning of a chase is likely indicated by increases in swimming velocity, dive depth, path tortuosity, and the production rate of echolocation clicks. Kinematic analysis of foraging dive behavior would therefore benefit from knowledge about how close a killer whale must be to a fish before it is energetically worth pursuing [84], as well as the threshold distance at which Pacific salmon are capable of detecting large predators. Echolocating killer whales can probably detect fish at depth during surface transits and initiate a foraging dive in response, as they are capable of sensing Chinook salmon at distances of up to 100 m in quiet conditions [84]. Construction of echograms from Dtag acoustic records to determine target ranges [85] and how these correspond to foraging movements would therefore also aid in identifying the sections of the water column important to killer whales for the search and initial pursuit phases of hunting.

The tortuous and non-linear swim paths exhibited by foraging killer whales (Fig. 2) support past field observations which indicated that, in addition to performing steep dives, salmon also attempt to avoid capture by unpredictably altering their swimming trajectories [86]. The smaller body size of salmon relative to that of killer whales allows them to execute tighter turning angles at faster rates, making them more maneuverable than their larger predators [68, 82, 87]. Evasive movements increase the probability of escape by taking the fish out of the direct pursuit path of the predator [82]. To intercept erratically swimming prey (and maintain knowledge of prey location using highly-directional echolocation clicks), killer whales must match the convoluted flight path of the fish. In our study, the response of tagged whales to the presumed evasive maneuvers of salmon resulted in lower straightness indices for foraging dives compared to respiration dives (Fig. 5, Table 3). However, mean straightness indices for foraging dives were similar to those of the ‘other’ dive category (Fig. 5, Table 3), indicating that measures of tortuosity alone may be insufficient to distinguish foraging from non-foraging behavior.

Rate of change in pointing angle was also expected to be noticeably higher during foraging dives compared to non-foraging dives (particularly for descents, where the majority of chasing behavior occurred), since the orientation of the whale’s longitudinal axis should change more rapidly in response to prey maneuvers. Although the rate of change in pointing angle for the training set of confirmed foraging dives (*N* = 15) was noticeably higher for both descents and ascents, this kinematic variable was roughly comparable across the three dive categories identified by the LDA (Table 3). This implies that other behaviors (e.g., socializing or beach-rubbing) also involve rapid orientation changes, which is supported by surface observations of resident killer whales. As expected, the change in pointing angle over time was generally higher for descents (chasing) than ascents (transiting to the surface); however, this was true for all dive types, not just foraging (Table 3). This finding implies that whales ascending from dives are likely returning to the surface using the most direct routes, probably because the need to replenish oxygen and off-load carbon dioxide take precedence over other activities near the end of a dive.

Higher swimming speeds exhibited by killer whales during foraging dives likely arose as a response to increased swimming speeds of fleeing prey. Unfortunately, very few studies have directly measured the maximum or burst swimming speeds of adult Pacific salmon in saltwater: the measure of swimming performance most relevant to avoiding predators [88]. Data logger measurements from a wild adult (4 winters at sea; European age 0.4) chum salmon in the Bering Sea measured a maximum speed of 2.8 m s^−1^ [53], which is comparable to the median speed recorded for successful killer whale foraging dives in our study (*M* = 2.7 m s^−1^, *N* = 15; Table 3). Randall et al. [89] determined the mean burst critical swimming speed (U_crit_ [90];) of Chinook salmon in saltwater to be 2.32 body lengths s^−1^, or about 0.731 m s^−1^ (sustained for 30–60 s). However, fish in that study were previously fatigued prior to determining burst U_crit_ and were relatively small (mean fork length = 31.5 cm). It is probable that the larger size classes of Chinook typically consumed by resident killer whales (mean fork lengths = 80.8–93.4 cm) [6] are capable of swimming much faster than this when pursued by whales. Killer whale maximum swim speeds were expected to approximate or marginally surpass those of Pacific salmon, since they are unlikely to expend additional energy by swimming faster than is required for prey capture.

## Conclusions

Using high-resolution accelerometer tags, we provide the first quantitative description of fine-scale foraging movements by resident killer whales hunting Pacific salmon. Increased dive depth, tortuosity, body roll, and estimated swimming velocity were determined to be the most useful kinematic measures for distinguishing foraging from other dive behaviors. Reconstructed dive paths indicated that foraging dives targeted the expected depth distribution of Chinook salmon, the preferred prey of resident killer whales, and whale movements during prey pursuit also revealed probable escape strategies used by salmon to avoid capture (rapid descent, evasive maneuvering, and increased swimming speeds). Future studies could build on our findings by using Dtag records to assess space use and energy expenditure by killer whales during different activity states. Our reconstructed Dtag tracks and the kinematic characteristics of foraging dives we have identified also provide a comparative baseline for evaluating the impacts of various anthropogenic disturbances on resident killer whale foraging behavior.

## Additional file

Additional file 1:
**Appendix A1.** Methods: Calculation of Kinematic Dive Variables. (DOCX 16 kb)

## Abbreviations

GPS: Global positioning system
IQR: Inter-quartile range
LDA: Linear discriminant analysis
M: Median
ODBA: Overall dynamic body acceleration
TDR: Time-depth recorder
VeDBA: Vectorized dynamic body acceleration
VHF: Very high frequency
