# Supplementary material for: Fine-scale foraging movements by fish-eating killer whales (Orcinus orca) relate to the vertical distributions and escape responses of salmonid prey (Oncorhynchus spp.)
Source: Mov Ecol. 2017 Feb 20;5:3. doi: 10.1186/s40462-017-0094-0 (PMC5319153; doi:10.1186/s40462-017-0094-0)
Supplement: Additional file 1: — Appendix A1. Methods: Calculation of Kinematic Dive Variables. (DOCX 16 kb) [file 40462_2017_94_MOESM1_ESM.docx]

**Additional file 1: Appendix A1**

**Methods: Calculation of Kinematic Dive Variables**

*Tortuosity (Path Straightness Index)*

Tortuosity is the degree of convolution in an animal’s path of movement, and was estimated using a simple straightness index [1-3]. We calculated this index as the ratio of the shortest (straight line) distance between two consecutive locations (*D*) and length of the dead-reckoned dive path (*L*) travelled by the whale between those two locations. Straightness (*D*/*L*) yields proportional values, with 1 representing a completely straight path and smaller values signifying a more convoluted, tortuous path. Using the dead-reckoned whale positions, we calculated straightness in both the two-dimensional, horizontal plane over whole dives and in three dimensions over each descent and ascent phase. Estimates of *D* and *L* were rounded to the nearest 0.1 m prior to calculating straightness. For two-dimensional straightness, the distance (*D*) portion of the equation was calculated using the Spherical Law of Cosines to determine the Great Circle distance between consecutive surfacing locations (where *φ*=latitude (rad), *λ*=longitude (rad), and *R*=radius of the Earth, or 6,371,000 m).

$$D=\arccos(\sin\left( \phi_{initial} \right) \times\sin\left( \phi_{final} \right)+\cos\left( \phi_{initial} \right)\times\cos\left( \phi_{final} \right) \times\cos(\Delta\lambda)) \times R$$

For three-dimensional straightness, the straight-line distances (*D*) of descents and ascents were calculated as the Euclidean distance between each surfacing location and the coordinates at maximum dive depth using an extension of the Pythagorean Theorem (where *φ*=latitude (rad), *λ*=longitude (rad), *d*=depth (m)).

$$D= \sqrt{{(\Delta\phi)}^{2}+ {(\Delta\lambda)}^{2}+ {(\Delta d)}^{2}}$$

We determined path lengths (*L*) for the straightness index by first calculating the Euclidean distance travelled over each sampling interval (0.2 s) using the Pythagorean Theorem in either two or three dimensions. We then summed these distances over the entire time period of interest (i.e., whole dive, descent, or ascent) to obtain the total path length (*L*) for that period. We also obtained an approximate estimate of overall swimming speed (m s^-1^) for each dive by dividing the dead-reckoned, three-dimensional dive path length by the total dive duration. Vertical velocities, conversely, were based solely on sensor data rather than dead-reckoned tracks, and were calculated as the change in depth over time (m s^-1^) for each dive phase (descent or ascent).

*Dynamic Body Acceleration*

Dynamic Body Acceleration (DBA) is a measure that summarizes tri-axial body acceleration and is considered a proxy for an individual’s movement-based metabolic rate (i.e., its energy expenditure as indicated by rate of oxygen consumption, or *V*O_2_) [4-5]. We calculated VeDBA (the vector summation of DBA), rather than the more commonly used Overall Dynamic Body Acceleration (ODBA) because VeDBA is a better proxy for *V*O_2_ when tag orientation varies over time [4]. To obtain VeDBA, we derived the static acceleration for each axis by smoothing the raw accelerometer data using a running mean of 3 s, which is appropriate for species whose dominant stroke period does not exceed this value [6]. This includes killer whales, which have a mean stroke period of 2.3 s [7]. To estimate dynamic acceleration, we subtracted the static values from the raw values and then determined VeDBA by calculating the vector sum of the derived dynamic accelerations of all three axes [4]. The mean value of VeDBA over the duration of each dive was then calculated.

*Mean Change in Roll & Mean Change in Pointing Angle*

Mean change in roll and pointing angle are estimates of the rate of change in whale body orientation and were calculated using the methods of Miller et al. [8] with minor modifications. To eliminate negative values resulting from direction of body rotation, we calculated changes in roll orientation as absolute values, which we assumed could not exceed 180° within each 0.2 s sampling interval. Pointing angle combines pitch and heading orientations to describe the three-dimensional angle of the whale’s longitudinal axis [8]. We calculated the change in pointing angle ($\Delta\hat{\sigma}$, analogous to the central angle between two locations on the surface of a sphere) using the Spherical Law of Cosines with the substitution of pitch (*p*) and heading (*h*) for latitude and longitude, respectively.

$$\Delta\hat{\sigma}=\arccos(\sin\left( p_{initial} \right) \times\sin\left( p_{final} \right)+\cos\left( p_{initial} \right)\times\cos\left( p_{final} \right) \times\cos(\Delta h))$$

Both change in roll and change in pointing angle were calculated for each sampling interval (0.2 s) of the calibrated data and smoothed by taking the running sum over 1 s intervals. We down-sampled the smoothed data from 5 Hz to 1 Hz before determining the mean change in roll and pointing angle (degree s^-1^) for the descent and ascent phases of every dive.

**References**

1. Benhamou S. How to reliably estimate the tortuosity of an animal's path: straightness, sinuosity, or fractal dimension? J Theor Biol. 2004;229:209-20.

2. Miller C, Christman MC, Estevez I. Movement in a confined space: Estimating path tortuosity. Appl Anim Behav Sci. 2011;135:13-23.

3. Weimerskirch H, Bonadonna F, Bailleul F, Mabille G, Dell'Omo G, Lipp H-P. GPS tracking of foraging albatrosses. Science. 2002;295:1259.

4. Qasem L, Cardew A, Wilson A, Griffiths I, Halsey LG, Shepard ELC et al. Tri-axial dynamic acceleration as a proxy for animal energy expenditure; should we be summing values or calculating the vector? PLoS ONE. 2012;7(2):1-8.

5. Wilson RP, White CR, Quintana F, Halsey LG, Liebsch N, Martin GR et al. Moving towards acceleration for estimates of activity-specific metabolic rate in free-living animals: the case of the cormorant. J Anim Ecol. 2006;75(5):1081-90.

6. Shepard ELC, Wilson RP, Halsey LG, Quintana F, Gómez Laich A, Gleiss AC et al. Derivation of body motion via appropriate smoothing of acceleration data. Aquat Biol. 2008;4:235-41.

7. Sato K, Watanuki Y, Takahashi A, Miller PJO, Tanaka H, Watanabe Y et al. Stroke frequency, but not swimming speed, is related to body size in free-ranging seabirds, pinnipeds, and cetaceans. Proc R Soc B. 2007;274:471-7.

8. Miller PJO, Johnson M, Tyack P. Sperm whale behaviour indicates the use of echolocation click buzzes 'creaks' in prey capture. Proc R Soc B. 2004;271:2239-47.
